# Ellagitannin Digestion in Moth Larvae and a New Dimeric Ellagitannin from the Leaves of *Platycarya strobilacea*

**DOI:** 10.3390/molecules26144134

**Published:** 2021-07-07

**Authors:** Juri Takayoshi, Yong-Lin Huang, Yosuke Matsuo, Yoshinori Saito, Dian-Peng Li, Takashi Tanaka

**Affiliations:** 1 Graduate School of Biomedical Sciences, Nagasaki University, 1-14 Bunkyo-machi, Nagasaki 852-8521, Japan; bb55621018@ms.nagasaki-u.ac.jp (J.T.); y-matsuo@nagasaki-u.ac.jp (Y.M.); saiyoshi@nagasaki-u.ac.jp (Y.S.); 2 Guangxi Key Laboratory of Functional Phytochemicals Research and Utilization, Guangxi Institute of Botany, Guilin 541006, China; ldp@gxib.cn

**Keywords:** insect tea, *Platycarya strobilacea*, ellagic acid, brevifolin carboxylic acid, gallic acid, platycaryanin E, ellagitannin

## Abstract

Ellagitannins (ETs) are plant polyphenols with various health benefits. Recent studies have indicated that the biological activities of ETs are attributable to their degradation products, including ellagic acid and its gut microflora metabolites, such as urolithins. Insect tea produced in the Guangxi region, China, is made from the frass of moth larvae that feed on the ET-rich leaves of *Platycarya strobilacea*. Chromatographic separation of the Guangxi insect tea showed that the major phenolic constituents are ellagic acid, brevifolin carboxylic acid, gallic acid, brevifolin, and polymeric polyphenols. Chemical investigation of the feed of the larvae, the fresh leaves of *P. strobilacea*, showed that the major polyphenols are ETs including pedunculagin, casuarictin, strictinin, and a new ET named platycaryanin E. The new ET was confirmed as a dimer of strictinin having a tergalloyl group. The insect tea and the leaves of *P. strobilacea* contained polymeric polyphenols, both of which were shown to be composed of ETs and proanthocyanidins by acid hydrolysis and thiol degradation. This study clarified that Guangxi insect tea contains ET metabolites produced in the digestive tract of moth larvae, and the metabolites probably have higher bioavailabilities than the original large-molecular ETs of the leaves of *P. strobilacea*.

## 1. Introduction

Tannins are a class of plant polyphenols that precipitate proteins [1] and are attracting increasing attention as food constituents with potential health benefits [2]. Tannins are chemically grouped into two major classes: condensed tannins (also called proanthocyanidins) and hydrolyzable tannins, and the hydrolyzable tannins include gallotannins and ellagitannins (ETs). ETs are esters of hexahydroxydiphenoyl (HHDP) or its biogenetically related acyl groups typically bounded to glucose [3,4,5]. Compared to the condensed tannins and gallotannins, ETs exhibit a large structural diversity associated with structures and location of the acyl groups as well as their molecular sizes. Similar to other tannins, interaction of ETs with salivary proteins by hydrophobic association causes unfavorable bitter and astringent taste [6]. The tannin–protein interaction also causes inhibition of digestive enzymes of herbivores, reducing the nutritional value of plants consumed as food [7]; therefore, ETs are thought to be plant defense substances, and the ecological significance of ETs and condensed tannins has long been discussed [8,9,10]. Common foods that contain ETs include raspberry, strawberry, pomegranate, and walnut, and recent studies have suggested that many of the biological activities of these foods are attributable to ETs. Oak wood is also rich in ETs; therefore, wine, whiskey, brandy, and other alcoholic beverages that are aged in oak barrels also contain ETs or their degradation products that originate from the oak wood [11,12]. ETs are large molecules (larger than 500 Da), and some are larger than 2000 Da [1,3,4,5]. Thus, ETs are not directly absorbed from the gastrointestinal tract. However, ETs show biological activities by oral administration, and it is now commonly accepted that the biological activities of ETs are attributable to their metabolites produced by intestinal microbiota [13,14], as with the case of tea catechins and proanthocyanidins [15]. Initial degradation of ETs by intestinal bacteria probably occurs via hydrolysis of the HHDP groups to give ellagic acid (**2**). Subsequent reductive metabolism of **2** by intestinal bacteria generates urolithins. Urolithin A (3,8-dihydroxy-6*H*-dibenzo[*b*,*d*]pyran-6-one, i.e., castoreum pigment) was originally found in exudate from beavers [16] and in renal calculi of sheep [17], suggesting that ET metabolism is common in herbivorous animals. Recent studies have confirmed the same metabolism of ETs in human intestinal microflora [13,14], and this has had a considerable impact on biological studies of ETs. From the viewpoint of chemical ecology, degradation of ETs by insects are also important; however, details of the degradation products are not sufficiently clarified [18,19]. In this study, we investigated insect tea produced in Southwest China that is made from the frass of moth larvae. Although there are several types of insect teas in China, the insect tea of the Guangxi region is made from the frass of larvae of *Hydrillodes* *morose* or *Nodaria niphona* fed on the ET-rich leaves of *Platycarya strobilacea* [20]. The purpose of this study was to reveal the ET-derived molecules in the insect tea and to clarify the metabolism of ETs in the insect digestive tract by comparing the constituents of the insect tea with the polyphenols in the larvae feed.

## 2. Results and Discussion

### 2.1. Constituents of Insect Tea

**Figure 1:**
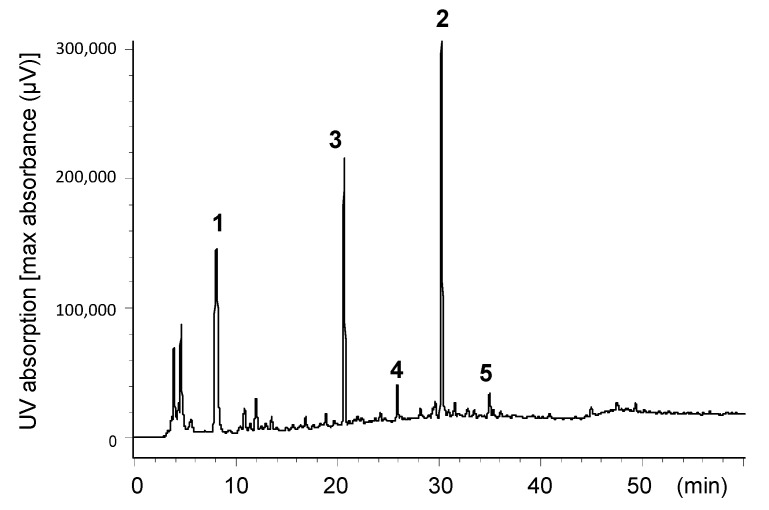
HPLC profile of insect tea: (**1**) Gallic acid; (**2**) ellagic acid; (**3**) brevifolin carboxylic acid; (**4**) brevifolin; (**5**) kaempferol 3-*O*-glucuronide.

**Figure 2:**
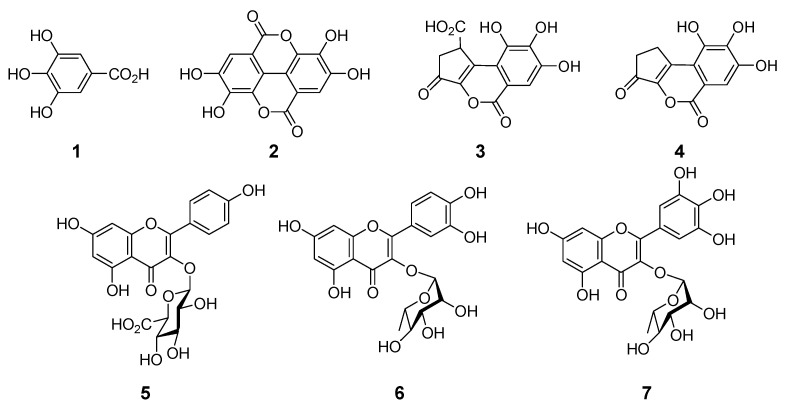
Structures of polyphenols isolated from insect tea: (**1**) Gallic acid; (**2**) ellagic acid; (**3**) brevifolin carboxylic acid; (**4**) brevifolin; (**5**) kaempferol 3-*O*-glucuronide; (**6**) quercetin 3-*O*-rhamnoside; and (**7**) myricitrin 3-*O*-rhamnoside.

Aqueous acetonitrile (CH_3_CN) extract of insect tea was analyzed by high-performance liquid chromatography (HPLC) with photodiode array detection (Figure 1). The chromatogram showed three prominent peaks, and the two at 8.1 min and 30.8 min were identified as gallic acid (**1**) and ellagic acid (**2**), respectively, by comparison of retention times and ultraviolet (UV) absorptions. To identify the remaining major compound observed at 20.8 min and other minor constituents, the aqueous acetone extract was separated on a large scale, and a comparison of nuclear magnetic resonance (NMR) data showed that the major compound detected at 20.8 min was brevifolin carboxylic acid (**3**) [21,22]. In addition, brevifolin (**4**) [23], kaempferol 3-*O*-glucuronide (**5**) [24], quercetin 3-*O*-rhamnoside (**6**) [25], and myricitrin 3-*O*-rhamnoside (**7**) [25] were isolated as the minor constituents (see Figure 2). The contents of **1**–**3** in the insect tea were estimated to be 1.6, 4.3, and 2.16 mg/g, respectively, by HPLC analysis. Furthermore, polymeric polyphenols detected at the origin on thin-layer chromatography (TLC) and observed as a broad hump on the HPLC baseline were obtained by size-exclusion column chromatography using Sephadex LH-20 with 7 M urea in acetone as the elution solvent [26].

The presence of **1** and **2** in the insect tea suggested that ETs in the feed (leaves of *P. strobilacea*) were degraded to acyl components in the gut of moth caterpillars. Ellagic acid (**2**) is derived from HHDP esters by hydrolysis. Production of **3** suggested the oxidative degradation of HHDP groups. It is known that the pH of midgut fluids of insect caterpillars is alkaline [18,19,27,28], and our previous study demonstrated that dehydrohexahydroxydiphenoyl (DHHDP) esters, the oxidized form of HHDP esters, decompose and generate **3** under weakly alkaline conditions [19,21].

### 2.2. Ellagitannins of Platycarya strobilacea

**Figure 3:**
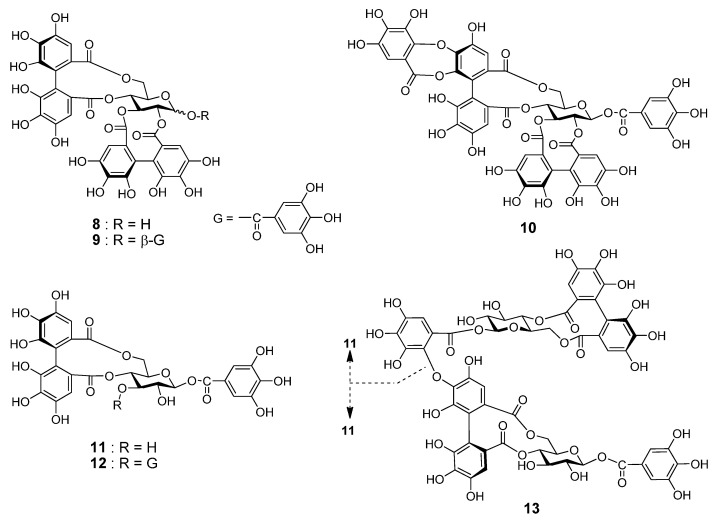
Structures of platycaryanin E (**13**) and known compounds pedunculagin (**8**), casuarictin (**9**), alnusnin A (**10**), strictinin (**11**), and 1,3-di-*O*-galloyl-4,6-(*S*)-HHDP-β-d-glucose (**12**). Platycaryanin E (**13**) is a dimer of strictinin (**11**).

The insect tea analyzed in this study was made by feeding moth larvae with the leaves of *Platycarya strobilacea*, a Juglandaceous deciduous tree distributed in Eastern Asia. The bark, wood, and fruit of *P*. *strobilacea* have been previously investigated and the presence of ETs, such as platycaryanins A–D, was reported [29,30,31]; however, the leaves have not been examined. Separation of aqueous acetone extract of the fresh leaves collected in July yielded six ETs. The major ET was pedunculagin (**8**) (0.55% from fresh leaves) [32], followed by casuarictin (**9**) (0.24%) [33], and alnusnin A (**10**) (0.21%) (Figure 3) [31,34]. Two of the minor ETs were identified as strictinin (**11**) and 1,3-di-*O*-galloyl-4,6-(*S*)-HHDP-β-d-glucose (**12**) [35]. The remaining ET (**13**) was found to be a new ET. In addition, the presence of catechin and kaempferol 3-*O*-glucuronide (**5**) was confirmed.

**Figure 4:**
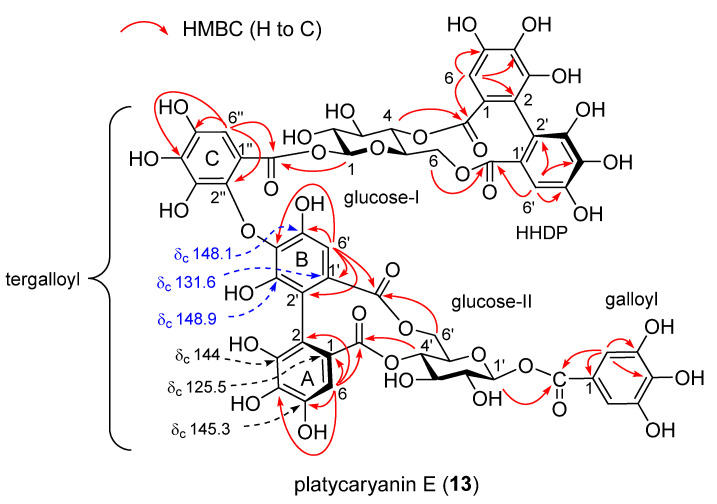
Selected NMR HMBC correlations and key ^13^C NMR signals of platycaryanin E (**13**).

Compound **13** was isolated as a brown amorphous powder and showed dark blue coloration after spraying with spraying iron(III) chloride (FeCl_3_) reagent on TLC. The ^13^C NMR spectrum showed signals arising from six pyrogallol-type aromatic rings accompanied by six ester carbonyl carbons. The dimeric nature of **13** was apparent from observation of two sets of ^1^H and ^13^C NMR signals of hexoaldoses, and it was confirmed by high-resolution fast-atom bombardment mass spectrometry (HRFABMS), which showed the [M+Na]^+^ peak at *m/z* 1289.1352 indicating the molecular formula of C_54_H_42_O_36_ (calcd. for C_54_H_42_O_36_Na: 1289.1348). The large coupling constants (8.2–9.9 Hz) of the aldose ring protons observed in the ^1^H NMR and ^1^H–^1^H COSY spectra revealed that both of the two aldoses were β-glucoses. The chemical shifts of the anomeric protons (δ_H_ 5.53 (glc-1) and 5.75 (glc-1’)), two C-4 methine protons (δ_H_ 4.79 (glc-4) and 4.94 (glc-4’)), and two-sets of C-6 methylene protons (δ_H_ 5.14 and 3.68 (glc-6), 5.14 and 3.78 (glc-6′)) demonstrated acylation of hydroxy groups at these positions of both glucoses. Furthermore, large differences of the chemical shifts of C-6 methylene proton signals were similar to those observed for **11**, suggesting formation of macrocyclic rings by acylation of HHDP groups at the glucose C-4 and C-6 hydroxy groups of glucopyranoses I and II [36]. This was supported by HMBC correlations of ester carbonyl carbons with aromatic protons and glucose ring protons (Figure 4).

As for the acyl groups, the presence of a galloyl group is apparent from the two-proton singlet at δ_H_ 7.18 in the ^1^H NMR spectrum and carbon signals at δ_C_ 110.2, 120.6, 139.4, 146.0, and 165.3 in the ^13^C NMR spectrum. Similarly, comparison of the NMR signals with those of **11** revealed the presence of a HHDP group (δ_H_ 6.67 (s, H-6) and 6.52 (s, H-6’)). Connection of the galloyl group to C-1 of glucose II and the HHDP group to glucose C-4 and C-6 of glucose I was shown by observation of the HMBC correlations between pyranose ring protons and aromatic singlets to corresponding ester carbonyls (Figure 4). The remaining acyl groups that connected glucoses I and II were suggested to be a gallic acid trimer with three isolated aromatic protons (δ_H_ 7.00 (s, H-6”), 6.72 (s, H-6), and 6.51 (s, H-6’)). There are three acyl groups composed of three galloyl components; they are tergalloyl [31], valoneoyl [37], and macaranoyl groups [38], which differ in the oxygen atom of the HHDP moiety where a galloyl group forms an ether linkage (tergalloyl, B-ring C-4’; valoneoyl, C-5’; and macaranoyl, C-3’). Among them, the galloyl group of **13** was determined to be the tergalloyl group based on appearance of the carbon signals at δ_C_ 131.6 (C-1’), 148.1 (C-5’), and 148.9 (C-3’) characteristic to the tergalloyl B-ring (Figure 4). In particular, the strong low-field shift of C-1’ at δ_C_ 131.6 as compared with those of the tergalloyl ring A (δ_C_ 125.5) and the HHDP esters (δ_C_ 126.6 and 127.0) was conclusive evidence for the identification. The location of the tergalloyl group was determined based on the HMBC correlations of sugar protons and aromatic protons to ester carbonyl carbons (Figure 4). In addition, a large positive Cotton effect at 238 nm and a negative Cotton effect at 261 nm indicated that the axial chirality of the two biphenyl bonds of the tergalloyl and HHDP groups are in *S* configuration [39]. These spectroscopic results allowed us to determine the structure of **13** as shown in Figure 3, and this compound was named platycaryanin E.

### 2.3. Polymeric Polyphenols

The Guangxi insect tea contained polymeric polyphenols detected as a dark-blue spot at the origin of the silica gel TLC with FeCl_3_ reagent. By HPLC analysis, the polymer was observed as a broad hump on the chromatogram baseline. Similar polymeric polyphenols were also found in the fresh leaves of *P. strobilacea*. The ^13^C NMR spectra of these polymeric polyphenols (Figure S1, Supplementary Materials) were related to each other and showed broad signals, suggesting the presence of procyanidins and ellagitannins. Signals in the range of δ_C_ 150–160 were assignable to proanthocyanidin A-ring C-5, 7, and 8a, and other aromatic carbon signals were attributed to pyrogallol- and catechol-type rings. In addition, ester carbon signals were observed in the range of δ_C_ 162–170, and the presence of sugar moieties was indicated by anomeric carbon signals at δ_C_ 95 and aliphatic carbon signals in the range of δ_C_ 62–78. To characterize the polymeric polyphenols chemically, thiol degradation for proanthocyanidins and acid hydrolysis for ellagitannins were applied to both samples. The thiol degradation of proanthocyanidins using mercaptoethanol produced thioethers of flavan-3-ol units by nucleophilic substitution at C-4 positions (Figure S2) [40]. The HPLC analysis of the thiol degradation products of polymeric polyphenols from *P. strobilacea* leaves showed prominent peaks attributable to thioethers of epigallocatechin, epicatechin, and their galloyl esters (Figure S3D). However, the chromatogram of polymeric polyphenols of insect tea showed only small peaks of thioethers and a large part of the broad hump arising from the polymers remained (Figure S3B). This result suggested that the original proanthocyanidins in the feed were converted to crosslinked substances in the digestive tract of the larvae and did not afford the usual thiol degradation products. However, acid hydrolysis of the two polymeric polyphenols yielded similar products and the major products were identified as gallic acid (**1**) and ellagic acid (**2**) (Figure S4). Therefore, the major components of the polymeric polyphenols of insect tea are considered to originate from the ETs in the leaves, and the polymers retain HHDP and galloyl groups.

### 2.4. Decomposition of Ellagitannins in the Digestive Tract of Moth Larvae

The results described above indicate that the galloyl and HHDP esters of the ETs of the feed are hydrolyzed to gallic acid (**1**) and ellagic acid (**2**) in the digestive tract of moth larvae. In addition, production of brevifolin carboxylic acid (**3**) and its decarboxylation product brevifolin (**4**) suggest that a part of the HHDP esters is oxidatively degraded in the larval gut, since the production mechanism of **3** from HHDP esters include the oxidative process [21]. Previously, Vihakas et al. showed that phenolic profiles of the frass of lepidopteran larvae fed on leaves containing ETs were similar to those of the alkali-treated extracts of the leaves, and they suggested that ETs were modified by the alkaline pH of the larval gut [19]. Because tannins are susceptible to autoxidation under alkaline conditions, **3** in the insect tea may by generated by autoxidation. More detailed investigations are necessary to clarify the production mechanism of **3** in the digestive tract.

## 3. Materials and Methods

### 3.1. General Information

Optical rotation was measured on a JASCO P-1020 digital polarimeter (JASCO, Tokyo, Japan). Fourier transform infrared (FTIR) spectra were measured on a JASCO FT/IR 410 spectrophotometer. The ultraviolet (UV) spectra were recorded using a JASCO V-560 UV/Vis spectrophotometer. Electronic circular dichroism (ECD) spectra were measured with a JASCO J-725N spectrophotometer. ^1^H and ^13^C NMR spectra were recorded on a Varian Unity Plus 500 spectrometer (Agilent, Santa Clara, CA, USA) operating at 500 and 126 MHz for ^1^H and ^13^C nuclei, respectively. ^1^H and ^13^C NMR spectra were also recorded on a JEOL JNM-AL400 spectrometer (JEOL, Tokyo, Japan) operating at 400 and 100 MHz for ^1^H and ^13^C nuclei, respectively. Coupling constants (*J*) were expressed in hertz and chemical shifts (δ) are reported in ppm with the solvent signal used as reference (acetone-*d*_6_: δ_H_ 2.04, δ_C_ 29.8). HRFABMS were recorded on a JMS700N spectrometer (JEOL) using *m*-nitrobenzyl alcohol or thioglycerol as the matrix. Column chromatography was performed using Sephadex LH-20 (25–100 µm, GE Healthcare, Little Chalfont, UK), Diaion HP20PSS (Mitsubishi Chemical, Tokyo, Japan), Chromatorex ODS (Fuji Silysia Chemical, Kasugai, Japan), and Toyopearl butyl-650M (Tosoh, Tokyo, Japan) columns. TLC analyses were performed on 0.25 mm thick, precoated silica gel 60 F_254_ plates (Merck, Darmstadt, Germany) with toluene–ethyl formate–formic acid (1:7:1, *v*/*v*/*v*) and on 0.1 mm thick, pre coated cellulose F plates (Merck, Darmstadt, Germany) with 2% aqueous acetic acid. Spots were detected by illumination under UV light (254 nm), followed by spraying with 2% ethanolic FeCl_3_ or 5% H_2_SO_4_ solution, and then heating. Analytical HPLC was performed on a Cosmosil 5C18-ARII (Nacalai Tesque, Kyoto, Japan) column (250 × 4.6 mm, i.d.) with a gradient elution of 4–30% (39 min) and 30%–75% (15 min) CH_3_CN in 50 mM H_3_PO_4_ at 35 °C (flow rate, 0.8 mL/min and detection, JASCO photodiode array detector MD-2010).

### 3.2. Materials

Commercial insect tea produced from excrements of *Hydrillodes morose* fed on *P. strobilacea* in Liuzhou, Guangxi Zhuang Autonomous Region, China, was purchased in Guilin. The leaves of *Platycarya strobilacea* were collected in Hagi city, Yamaguchi Prefecture, Japan, in July 2020. The insect tea and voucher specimen of *P. strobilacea* were deposited at the Nagasaki University Graduate School of Biomedical Sciences.

### 3.3. HPLC Analysis

Insect tea (100 mg) was extracted with 75% CH_3_CN (2.0 mL) at 80 °C and analyzed by HPLC (Figure 1). Standard samples of **1**–**3** were obtained by acid hydrolysis or alkaline degradation of ellagitannins [21,22]. Briefly, 1-*O*-galloyl-2,3-(*R*)-dehydrohexahydroxydiphenoyl-3,6-(*R*)-HHDP-β-D-glucose (geraniin) was hydrolyzed in 2% sulfuric acid under reflux for 5 h. The fine needles formed in the reaction mixture were collected by filtration to give ellagic acid (**2**). The filtrate was separated by Sephadex LH-20 column chromatography with H_2_O containing increasing proportions of methanol (MeOH) to afford gallic acid (**1**). As for brevifolin carboxylic acid (**3**), geraniin was heated in 0.2% sodium hydroxide at 70 °C for 10 min. The mixture was acidified with 1% hydrochloric acid and separated by Sephadex LH-20 column chromatography (0–80% MeOH) to yielded **1**, **2**, and **3**.

### 3.4. Extraction and Separation

#### 3.4.1. Insect Tea

Insect tea (100 g) was suspended in H_2_O (250 mL) and heated at 80 °C for 15 min. After cooling, acetone (750 mL) was added and stirred at room temperature for 12 h. The suspension was filtered by suction filtration and the debris was extracted again with 75% acetone (300 mL) at room temperature for 12 h. The filtrates were combined, and acetone was evaporated under reduced pressure using a rotary evaporator. The insoluble substance in the resulting aqueous solution was removed by filtration, and the filtrate was applied to a Diaion HP20SS column (4 cm i.d. × 18 cm) with H_2_O containing increasing proportions of MeOH (0–100%, 5% stepwise, each 100 mL), and finally the column was washed with acetone. The eluate was collected by a fraction collector and each test tube was checked by TLC to give 6 fractions (fr.): fr. 1 (0.3 g), fr. 2 (0.4 g), fr. 3 (0.35 g), fr. 4 (0.86 g), fr. 5 (0.79 g), and fr. 6 (1.21 g). The major component of frs. 1 and 2 was identified as gallic acid (**1**) by TLC and HPLC. Fraction 4 was separated by Sephadex LH-20 column chromatography (3 cm i.d. × 20 cm) with H_2_O containing increasing proportions of MeOH (0–100%, 10% stepwise, each 50 mL) to give brevifolin carboxylic acid (**3**) (24.8 mg) as yellow precipitate from H_2_O. Fraction 5 contained mainly polymeric polyphenols and was separated by size-exclusion column chromatography using Sephadex LH-20 (2 cm i.d. × 55 cm) with 7 M urea–acetone (2:3, *v*/*v*, conc. HCl 2 mL/L). The fractions containing polymeric polyphenols, which were detected at the origin of silica gel TLC, were combined and concentrated to remove acetone. To remove urea and HCl, the aqueous solution was applied to a Diaion HP20SS column (2 cm i.d. × 15 cm) with H_2_O containing increasing proportions of MeOH (0–80%, 10% stepwise, each 20 mL) to yield polymeric polyphenols (312.9 mg). The fractions of the size-exclusion column chromatography containing low-molecular weight polyphenols was also passed through a Diaion HP20SS column to give fr. 5-2 (401 mg). Fraction 6 was suspended in 30% acetone solution at room temperature for 18 h and the pale-yellow precipitate was collected by filtration. The precipitates were washed with aqueous acetone to give ellagic acid (**2**) (172 mg). The filtrate was separated on a Sephadex LH-20 column (3 cm i.d. × 20 cm) with H_2_O containing increasing proportions of MeOH (0–100%, 10% stepwise, each 100 mL) to give six subfractions. Fraction 6-3 (224 mg) was applied to a Chromatorex ODS column (2 cm i.d. × 15 cm) with H_2_O containing increasing proportions of MeOH (0–100%, 5% stepwise, each 50 mL) to give brevifolin (**4**) (19.1 mg). Fraction 6-4 (257 mg) was applied to a Chromatorex ODS column (2 cm i.d. × 15 cm) with H_2_O containing increasing proportions of CH_3_CN (0–80%, 5% stepwise, each 50 mL) to give myricitrin 3-*O*-rhamnoside (**7**) (9.1 mg), kaempferol 3-*O*-glucuronide (**5**) (3.4 mg), and quercetin 3-*O*-rhamnoside (**6**) (8.7 mg) (Chart S1, Supplementary Materials).

#### 3.4.2. *Platycarya strobilacea*

Fresh leaves of *P. strobilacea* (450 g) were extracted with 70% aqueous acetone (3 L × 2). The extract was concentrated, and the resulting insoluble precipitates were removed by filtration. The filtrate was applied to a column of Diaion HP20SS (4 cm i.d. × 30 cm) with 0–100% MeOH (10% stepwise gradient elution, each 500 mL) to give two fractions: fr. 1 (22.5 g) and fr. 2 (12.7 g). Separation of fr. 1 on Sephadex LH-20 (4 cm i.d. × 30 cm, 0–100% MeOH) gave polymeric polyphenols (fr. 1-1, 661 mg), fr. 1-2 (197 mg), fr. 1-3 (10.5 g), and fr. 1-4 (11.8 g). HPLC analysis of fr. 1-2 and fr. 2 indicated the presence of kaempferol 3-*O*-glucuronide (**5**) in these fractions. Fraction 1-3 was subjected to a combination of chromatography using Diaion HP20SS, Chromatorex ODS, Toyopearl butyl 650M, and Sephadex LH-20 to yield pedunculagin (**8**) (2.48 g), strictinin (**11**) (117 mg), catechin (253.6 mg), 1,3-di-*O*-galloyl-4,6(S)-HHDP-β-d-glucose (**12**) (31.9 mg), and platycaryanin E (**13**) (65.5 mg). Fraction 1-4 was subjected to Diaion HP20SS column chromatography (4 cm i.d. × 27 cm, 0–100% MeOH) to give alnusnin A (**10**) (927 mg) and casuarictin (**9**) (1.10 g) (Chart S2, Supplementary Materials).

### 3.5. Spectroscopic Data

#### Platycaryanin E (**13**)

Brown amorphous powder, [α]_D_ −45.3 (*c* 0.1, MeOH). IR ν_max_ cm^−1^: 3419, 2998, 2932, 1730, 1624, 1515, 1453, 1338, 1201, and 1051. UV λ_max_ (MeOH) nm (log ε): 205 (5.00), 269 (4.58). HRFABMS *m/z*: 1289.1352 (calcd. for C_54_H_42_O_36_Na: 1289.1348). ECD (MeOH) Δε (nm): 0 (211), +39.9 (238), 0 (251), −17.5 (261), 0 (274), +5.3 (282), 0 (293), −4.2 (312). ^1^H NMR (acetone-*d*_6_, 500 MHz) δ_H_: 7.18 (s, galloyl-2,6), 7.00 (s, tergalloyl-6”), 6.72 (s, tergalloyl-6), 6.67 (s, HHDP-6), 6.52 (s, HHDP-6’), 6.51 (s, tergalloyl-6’), 5.75 (d, *J* = 8.2 Hz, glc-1’), 5.53 (d, *J* = 8.3 Hz, glc-1), 5.14 (dd, *J* = 6.4, 13.2 Hz, glc-6’), 5.11 (dd, *J* = 6.4, 13.7 Hz, glc-6), 4.94 (t, *J* = 9.9 Hz, glc-4’), 4.79 (t, *J* = 9.9 Hz, glc-4), 4.12 (dd, *J* = 6.4, 9.9 Hz, glc-5’), 4.08 (dd, *J* = 6.4, 9.9 Hz, glc-5), 3.87 (t, *J* = 9.9 Hz, glc-3’), 3.78 (d, *J* = 13.2 Hz, glc-6’), 3.75 (t, *J* = 9.9 Hz, glc-3), 3.70 (dd, *J* = 8.2, 9.9 Hz, glc-2’), 3.68 (d, *J* = 13.7 Hz, glc-6), 3.61 (br t, *J* = 9.0 Hz, glc-2). ^13^C NMR (acetone-*d*_6_, 126 MHz) δ: 168.3 (tergalloyl-7), 168.1 (HHDP-7), 168.0 (tergalloyl-7’), 168.0 (HHDP-7’), 166.2 (tergalloyl-7”), 165.3 (galloyl-7), 148.9 (tergalloyl-3’), 148.1 (tergalloyl-5’), 146.0 (galloyl-3,5), 145.3 (tergalloyl-5), 145.0 (HHDP-5’), 145.0 (HHDP-5), 144.3, 144.2, 144.0 (HHDP-3, 3’, tergalloyl-3), 142.1 (tergalloyl-5’’), 141.9 (tergalloyl-2”), 140.0 (tergalloyl-4”), 139.7 (tergalloyl-3”), 139.4 (galloyl-4), 136.7 (tergalloyl-4), 136.4 (HHDP-4), 136.1 (tergalloyl-4’), 136.0 (HHDP-4’), 131.6 (tergalloyl-1’), 127.0, 126.6 (HHDP-1, 1’), 125.5 (tergalloyl-1), 120.7 (galloyl-1), 116.1 (HHDP-2), 115.8 (tergalloyl-2), 115.7 (HHDP-2’), 114.4 (tergalloyl-2’), 111.9 (tergalloyl-1’’), 110.2 (galloyl-2,6), 108.6 (tergalloyl-6”), 108.4 (tergalloyl-6’), 108.3 (tergalloyl-6), 108.2 (HHDP-6), 107.9 (HHDP-6’), 96.2 (glc-1’), 95.9 (glc-1), 75.8 (glc-3), 75.4 (glc-3’), 74.6 (glc-2’), 74.2 (glc-2), 73.3 (glc-5), 72.9 (glc-5’), 72.7 (glc-4’), 72.5 (glc-4), 64.1 (glc-6’), 63.6 (glc-6).

### 3.6. Thiol Degradation of Polymeric Polyphenols

Polymeric polyphenols obtained from insect tea and *P. strobilacea* (fr. 1-1) (each 5 mg) were separately dissolved in 60% ethanol (EtOH) (1.0 mL) containing 2-mercaptoethanol (40 μL) (Kanto Chemical, Tokyo, Japan) and concentrated HCl (5 μL) (Kishida Chemical, Osaka, Japan). The mixture was heated at 70 °C for 7 h and analyzed by HPLC. The standard thiol degradation products were obtained from persimmon proanthocyanidins [40].

### 3.7. Acid Hydrolysis of Polymeric Polyphenols

Polymeric polyphenols obtained from insect tea and *P. strobilacea* (fr. 1-1) (each 10 mg) were hydrolyzed by heating in 2% H_2_SO_4_ (1.0 mL) at 110 °C for 8 h in a screw-capped vial. After cooling, the mixture was diluted with EtOH (1.0 mL) and analyzed by HPLC. The chromatogram showed peaks attributable to gallic acid (8.9 min) and ellagic acid (30.7 min), which were identified by comparison of *t*_R_ and UV absorptions with those of authentic samples.

## 4. Conclusions

In summary, we compared ETs of *P. strobilacea* leaves with the ellagitannin-derived constituents in excrements of moth larvae fed on the leaves. The major ETs in the leaves are pedunculagin (**8**) and related compounds typically bearing HHDP and galloyl esters. The presence of gallic acid (**1**), ellagic acid (**2**) and brevifolin carboxylic acid (**3**) in the excrements showed occurrence of ester hydrolysis of ellagitannin acyl groups and further oxidative modification in the digestive tract of the moth larvae. Although further studies on the production mechanism of **3** are necessary, the findings of this study have implications for understanding the fate of ETs in nature. In addition, a new ellagitannin named platycaryanin E (**13**) was isolated from the leaves of *P. strobilacea,* and the structure was determined to be a dimer of strictinin (**11**) having a tergalloyl ester group.

## Data Availability

Not applicable.

## Supplementary Materials

The following are available online, Chart S1,S2: Flow charts of chromatographic separation, Figure S1: ^13^C NMR spectra of polymeric polyphenols obtained from insect tea (**A**) and *Platycarya strobilacea* (**B**) (DMSO-*d*_6_, 100 MHz), Figure S2: Thiol degradation of proanthocyanidins, Figure S3: HPLC of polymeric polyphenols and thiol degradation products, Figure S4: HPLC of acid hydrolysis products of polymeric polyphenols from insect tea (**A**) and polymeric polyphenols from *P. strobilacea* (**B**), Figure S5: ^1^H NMR spectrum of **13** (acetone-*d*_6_), Figure S6: ^13^C NMR spectrum of **13** (acetone-*d*_6_), Figure S7: ^1^H–^1^H COSY spectrum of **13** (acetone*-d*_6_), Figure S8: HSQC spectrum of **13** (acetone-*d*_6_), Figure S9: HMBC spectrum of **13** (acetone-*d*_6_), Figure S10: HRFABMS of **13**.

## Sample Availability

Samples of the compounds are not available from the authors.
