# Supplementary material for: Ellagitannin Digestion in Moth Larvae and a New Dimeric Ellagitannin from the Leaves of Platycarya strobilacea
Source: Molecules. 2021 Jul 7;26(14):4134. doi: 10.3390/molecules26144134 (PMC8303904; doi:10.3390/molecules26144134)
Supplement: Supplementary file 1 [file molecules-26-04134-s001.zip › molecules-1280379-supplementary.pdf]

Supplementary Materials

Ellagitannin Digestion in Moth Larvae and a New Dimeric Ellagitannin from the Leaves of *Platycarya strobilacea*

Juri Takayoshi [1], Yong-Lin Huang [2,*], Yosuke Matsuo [1], Yoshinori Saito [1], Dian-Peng Li [2] and TakashiTanaka [1,*]

[1] Graduate School of Biomedical Sciences, Nagasaki University, 1-14 Bunkyo-machi, Nagasaki 852-8521, Japan

[2] Guangxi Key Laboratory of Functional Phytochemicals Research and Utilization, Guangxi Institute of Botany, Guilin541006, China

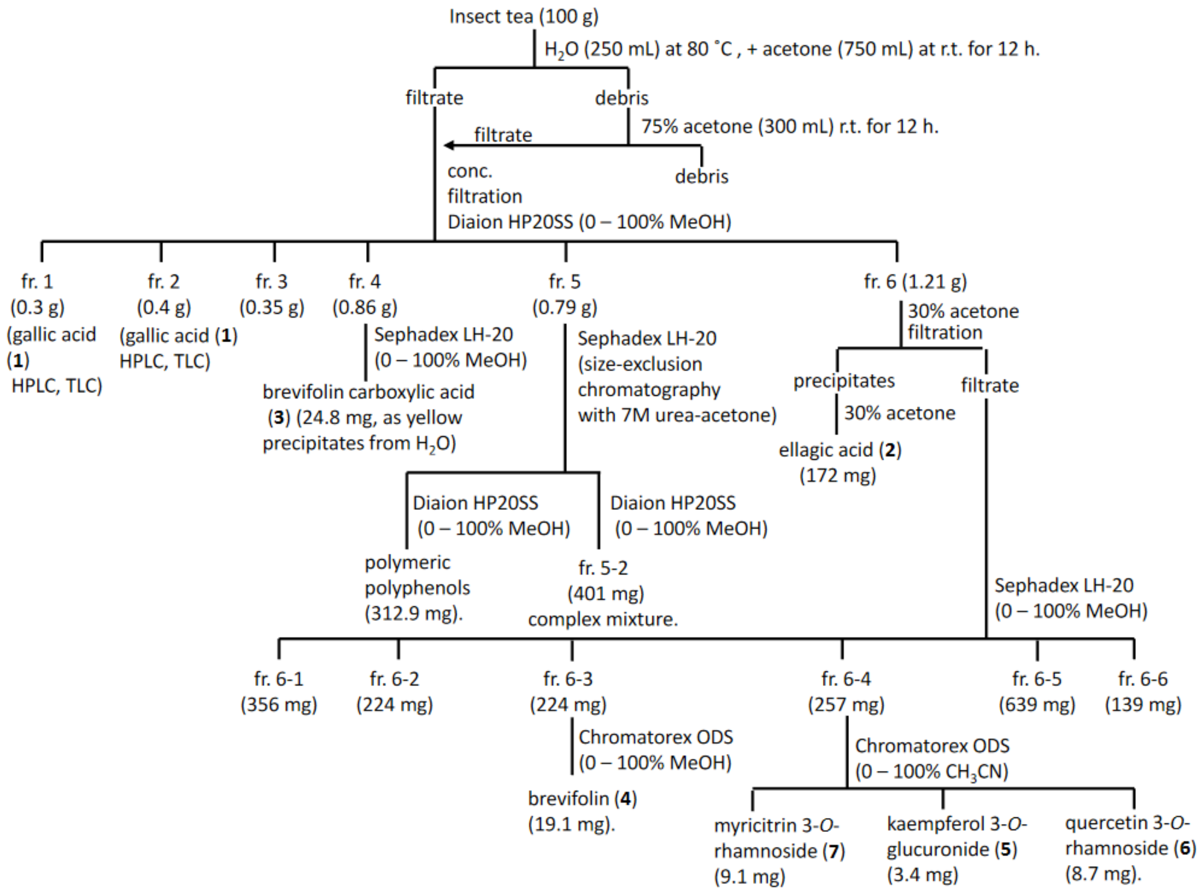

Chart S1. Separation flow chart of insect tea.

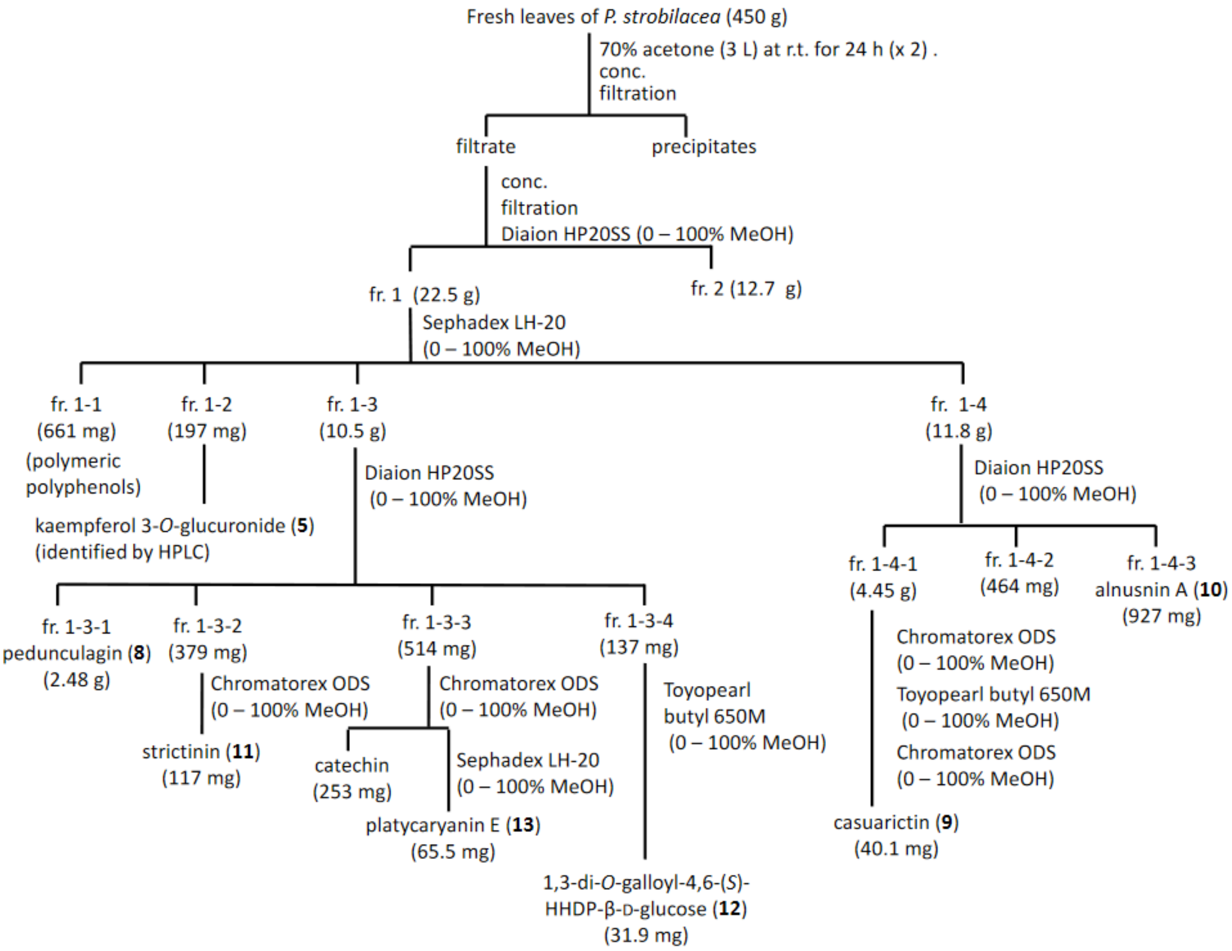

Chart S2. Separation flow chart of *P. strobilacea*.

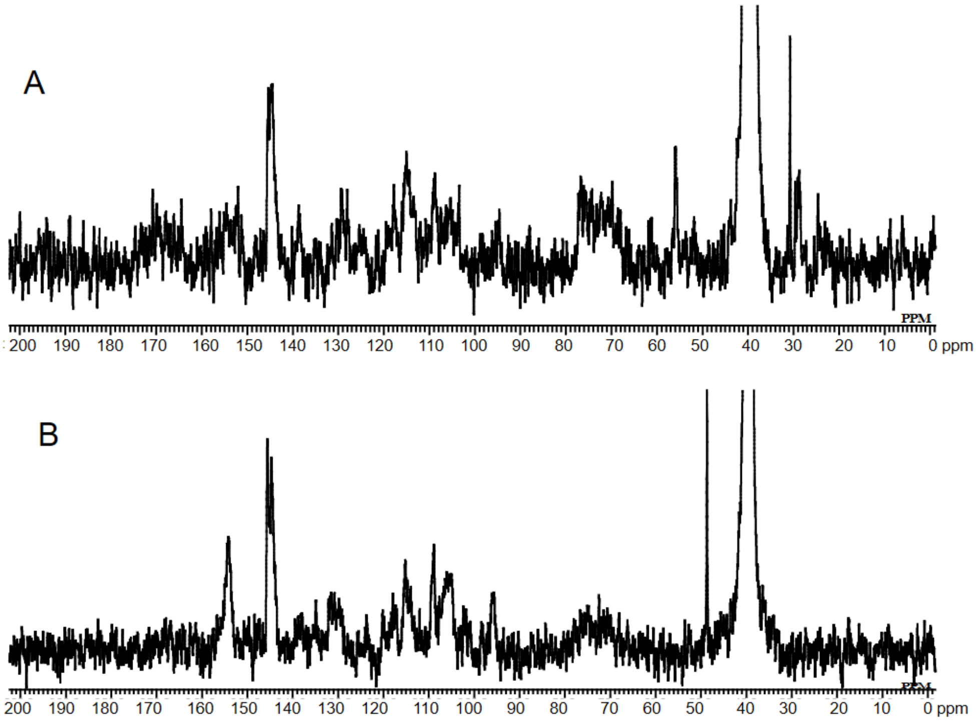

Figure S1. ^{13}C NMR spectra of polymeric polyphenols obtained from insect tea (**A**) and *P. strobilacea.* (**B**) (DMSO-d_6, 100 MHz).

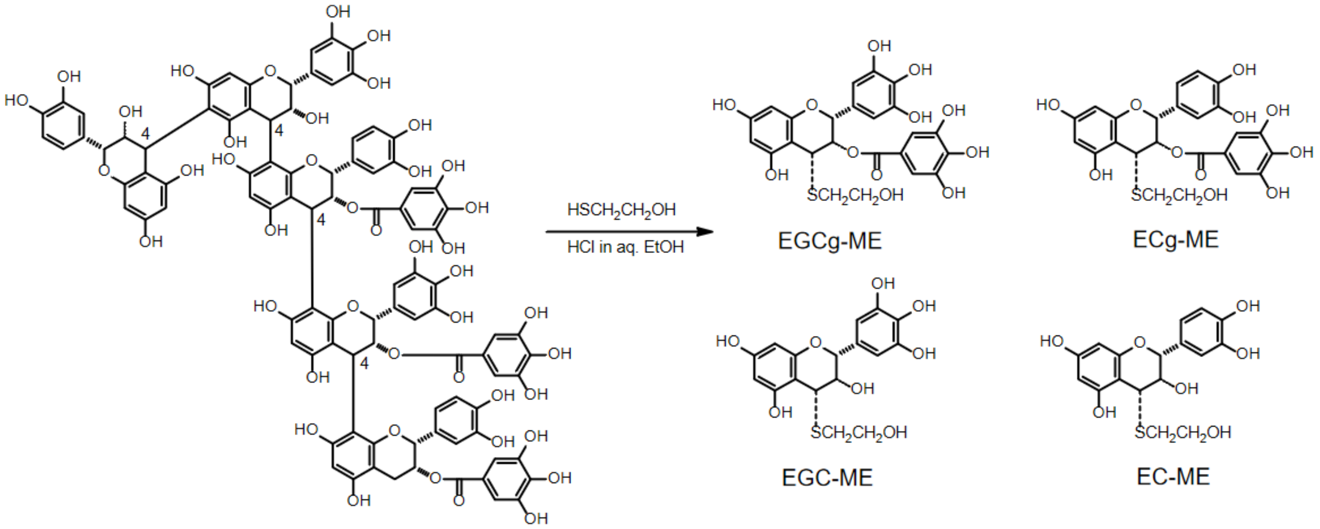

Figure S2. Thiol degradation of proanthocyanidins.

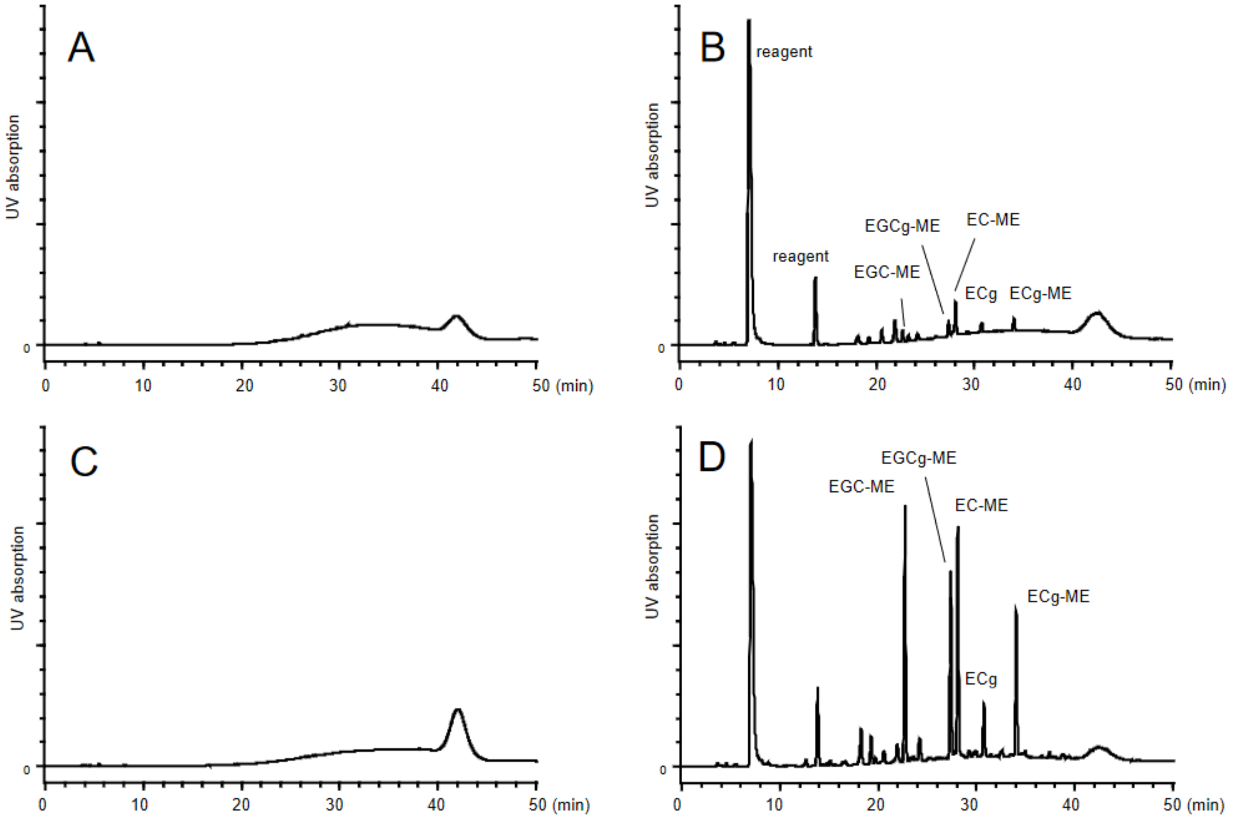

Figure S3. HPLC of polymeric polyphenols and the thiol degradation products.

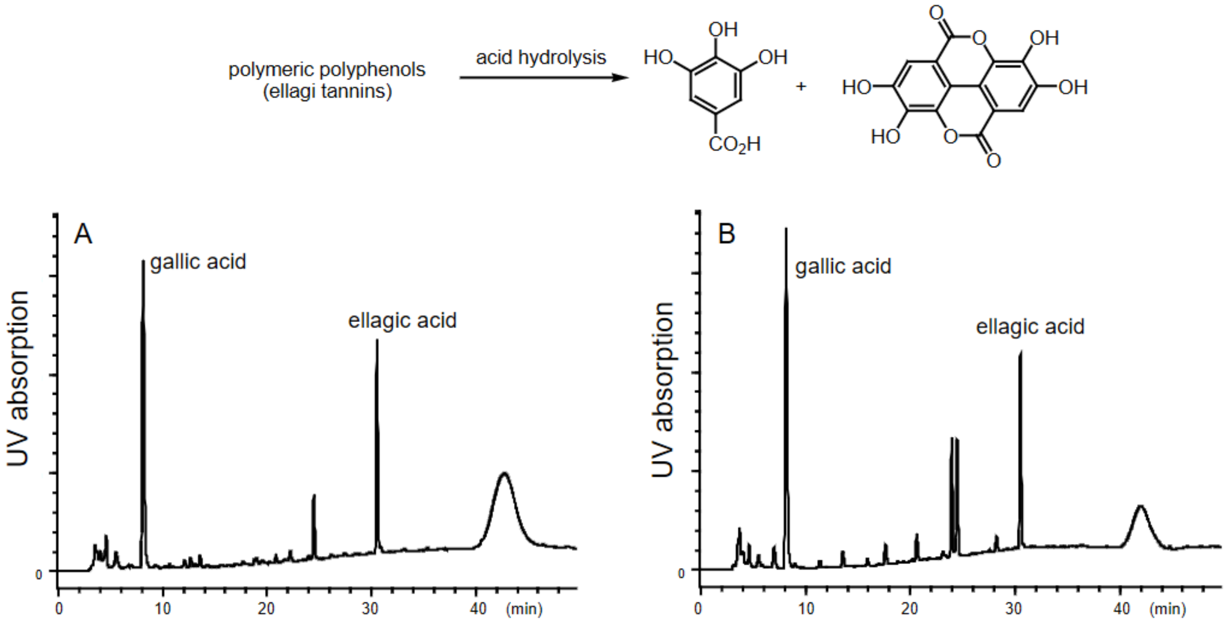

Figure S4. HPLC of acid hydrolysis products of polymeric polyphenols from insect tea (**A**) andpolymeric polyphenols from *P. strobilacea* (**B**).

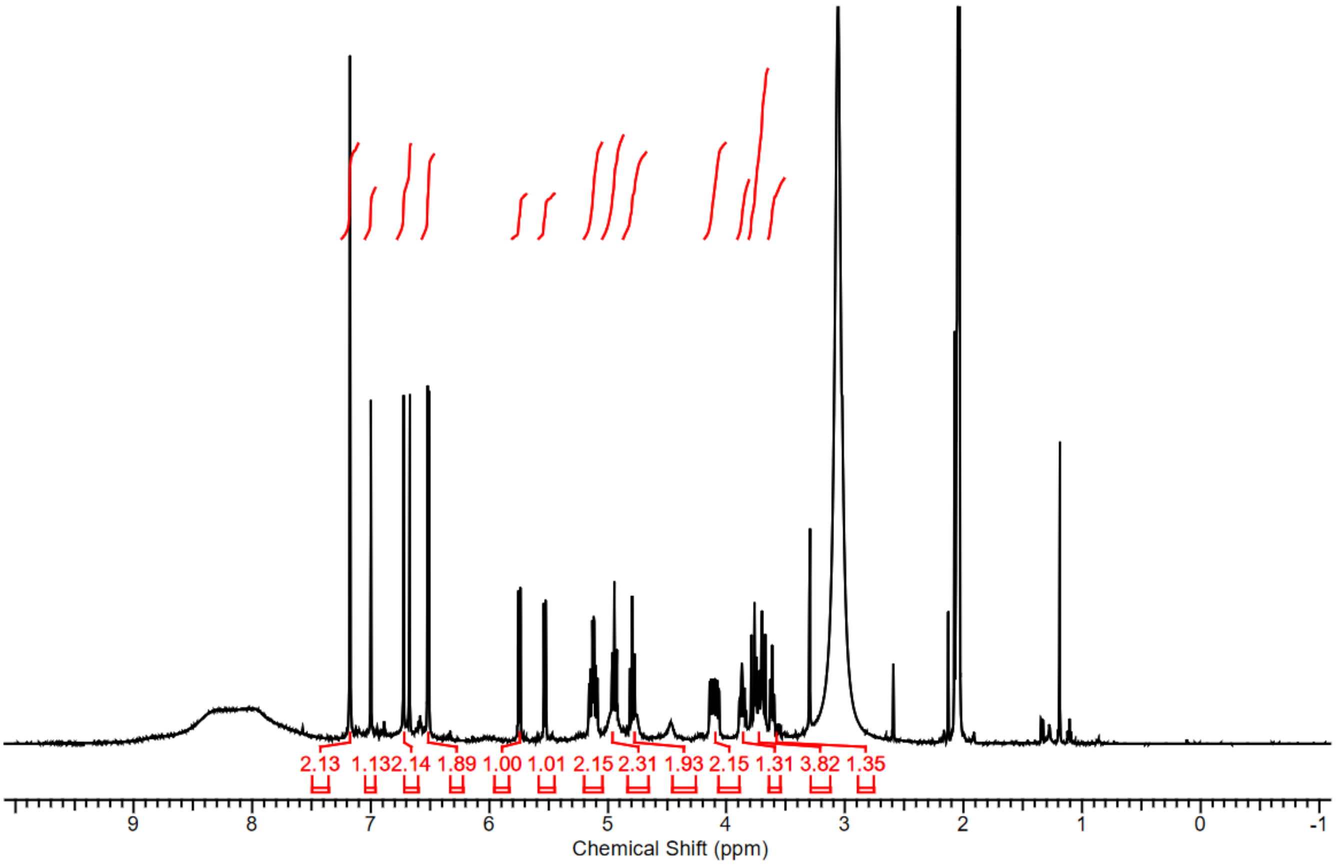

Figure S5. ^{1}H NMR spectrum of **13** (acetone-d_6).

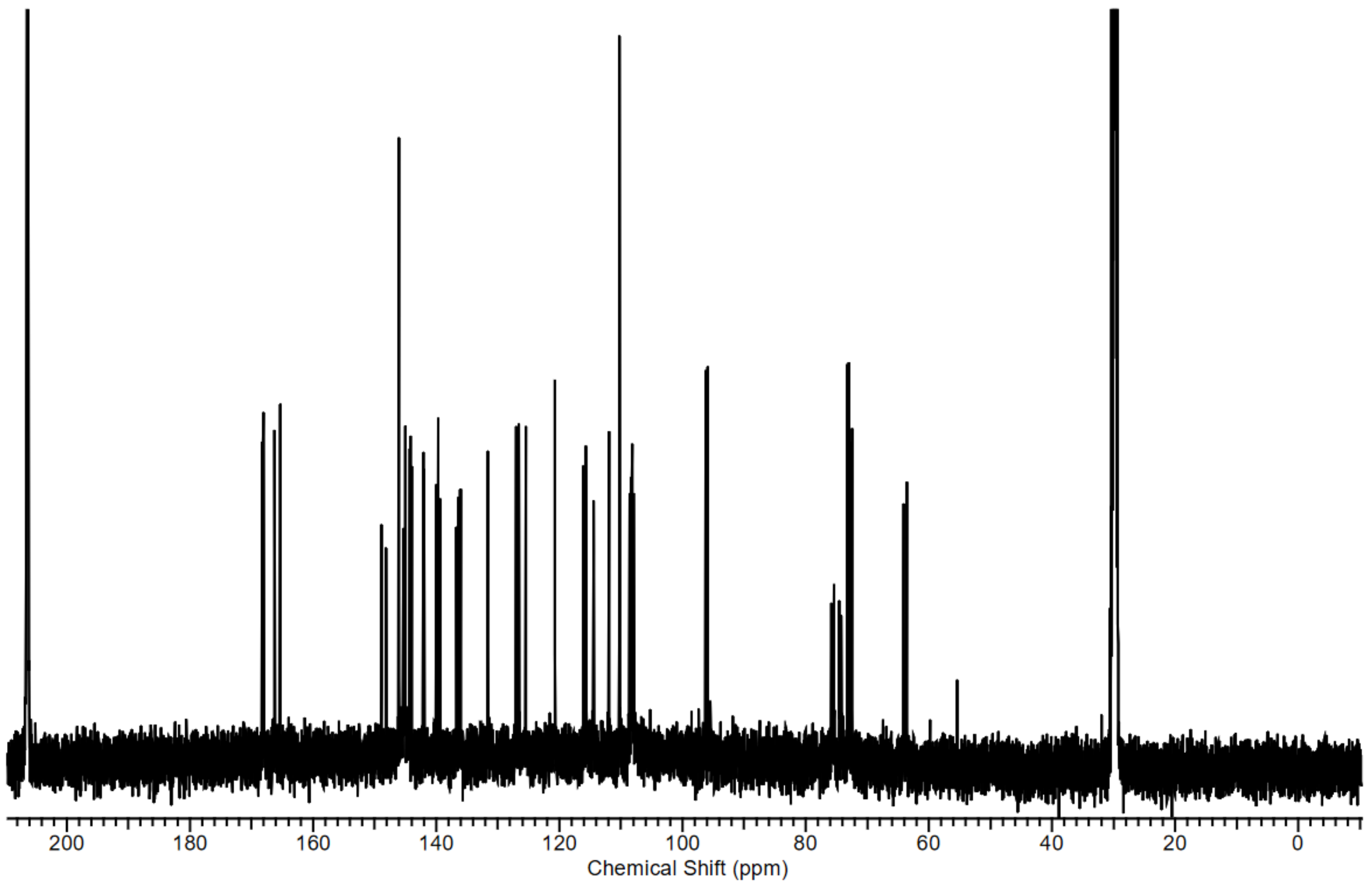

Figure S6. ^{13}C NMR spectrum of **13** (acetone-*d*$_6$).

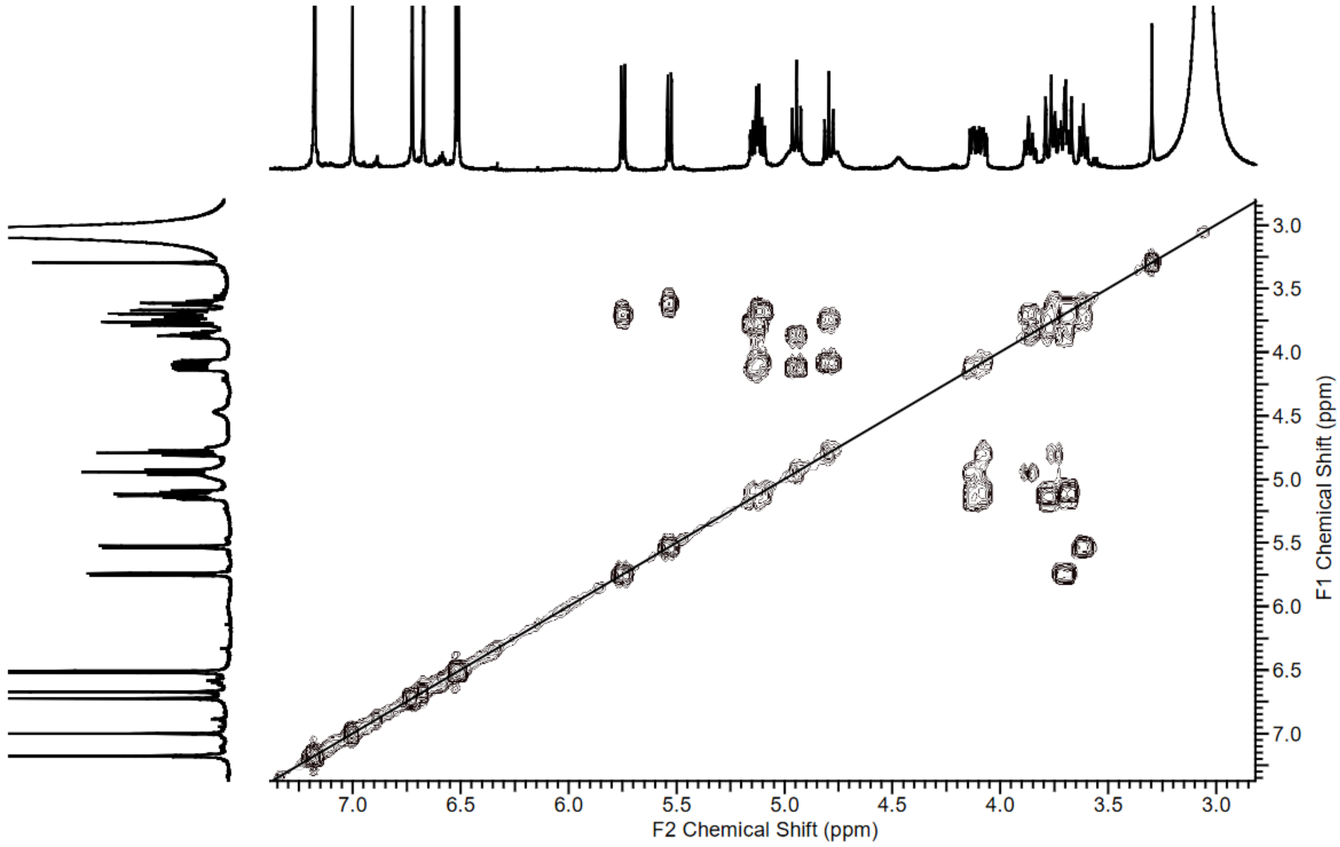

Figure S7. ^{1}H-^{1}H COSY spectrum of **13** (acetone-*d*$_6$).

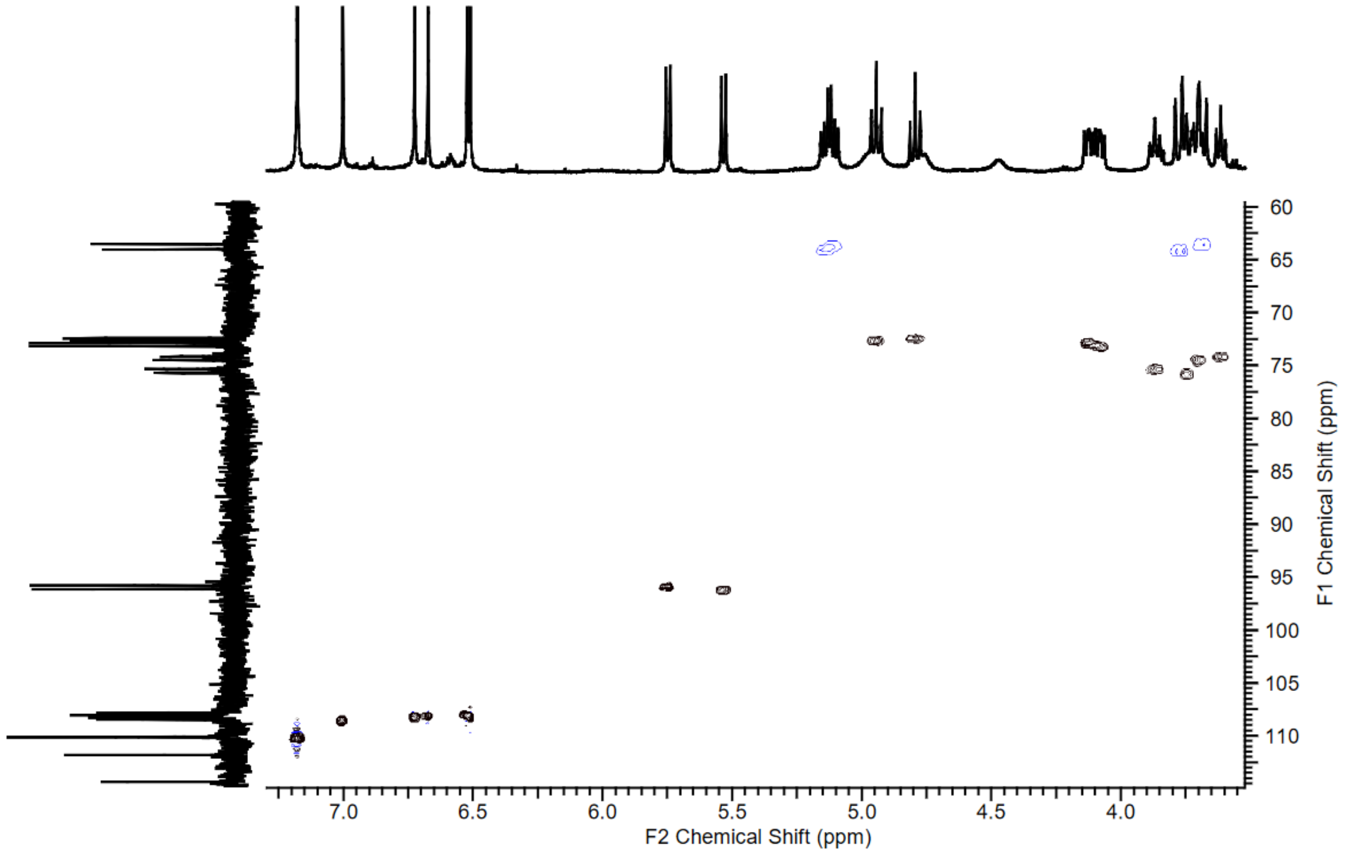

Figure S8. HSQC spectrum of **13** (acetone-d_6).

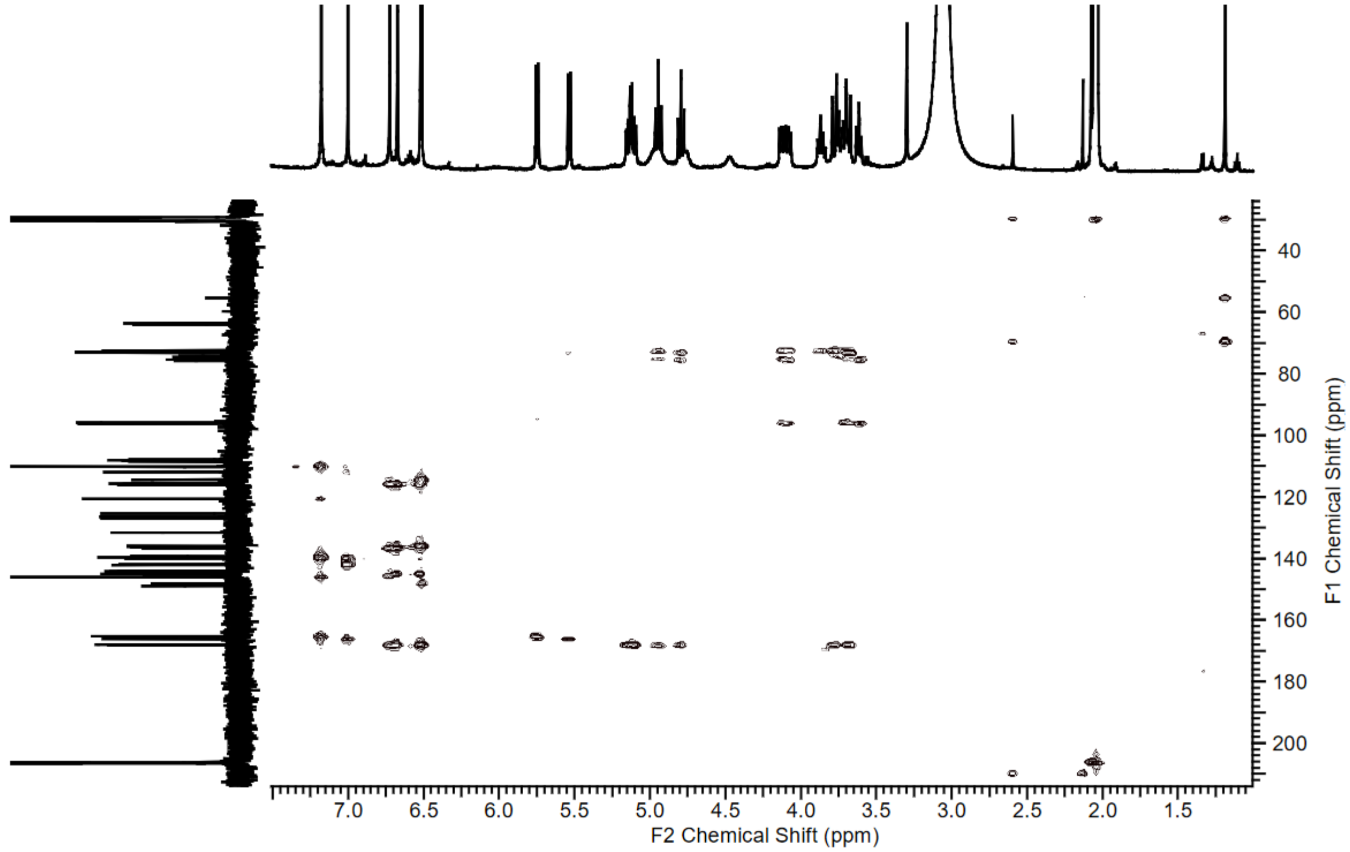

Figure S9. HMBC spectrum of **13** (acetone-d_6).

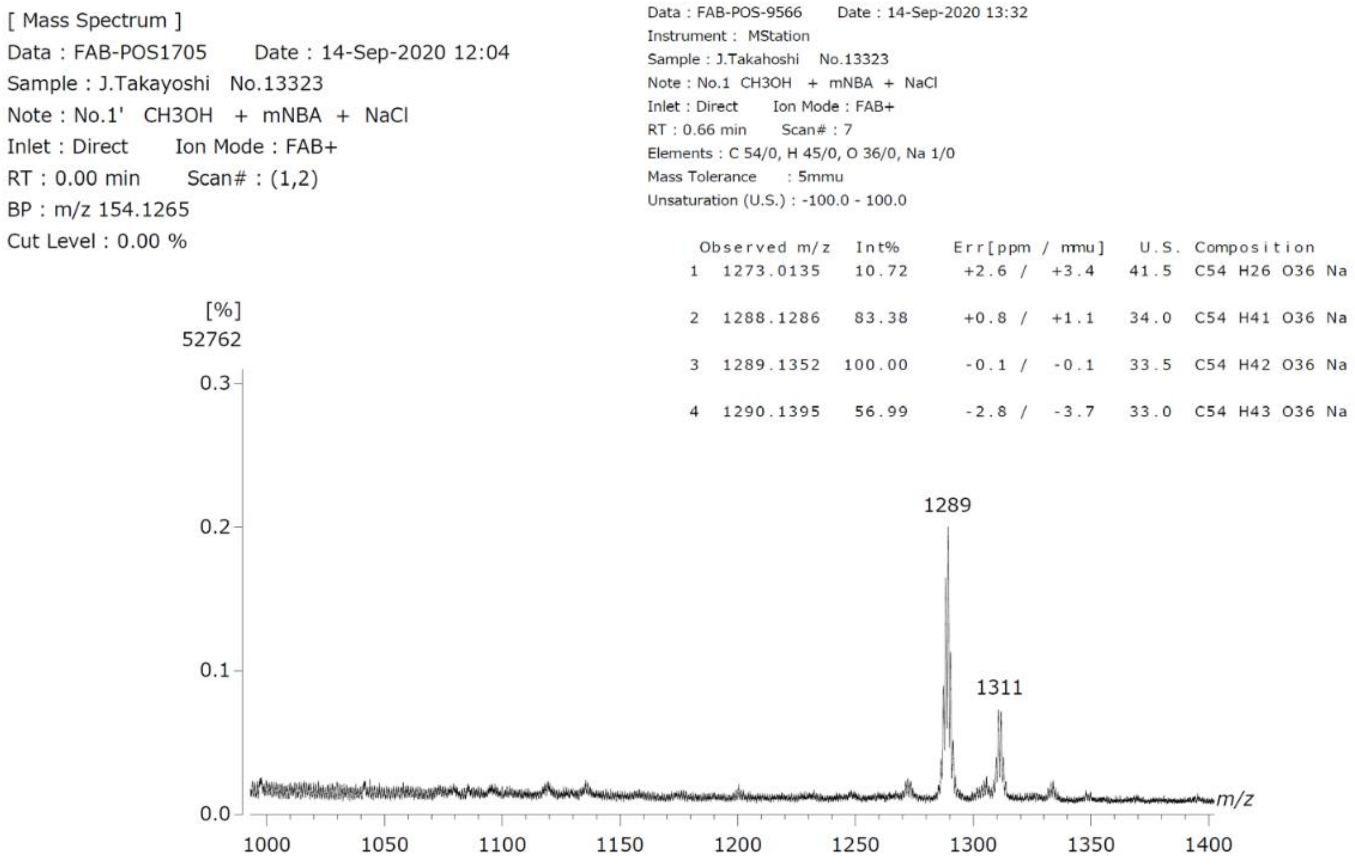

Figure S10. FAB-MS of 13.
